# Ultrahigh spin thermopower and pure spin current in a single-molecule magnet

**DOI:** 10.1038/srep04128

**Published:** 2014-02-19

**Authors:** Bo Luo, Juan Liu, Jing-Tao Lü, Jin-Hua Gao, Kai-Lun Yao

**Affiliations:** 1 Wuhan National High Magnetic Field Center, Huazhong University of Science and Technology, Wuhan 430074, People's Republic of China; 2 School of Physics, Huazhong University of Science and Technology, Wuhan 430074, People's Republic of China

## Abstract

Using the non-equilibrium Green's function (NEGF) formalism within the sequential regime, we studied ultrahigh spin thermopower and pure spin current in single-molecule magnet(SMM), which is attached to nonmagnetic metal wires with spin bias and angle (*θ*) between the easy axis of SMM and the spin orientation in the electrodes. A pure spin current can be generated by tuning the gate voltage and temperature difference with finite spin bias and the arbitrary angle except of 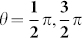. In the linear regime, large thermopower can be obtained by modifying *V_g_* and the angles (*θ*). These results are useful in fabricating and advantaging SMM devices based on spin caloritronics.

Studies on nanoscale thermoelectric devices have attracted much attention during the past a few years^1^,^2^,^3^,^4^. It is well accepted that nanoscale materials may provide an opening for the thermoelectricity in meeting the challenge of being a sustainable energy source^5^. Huge deviation from the Wiedemann-Franz law^6^,^7^ in the nanostructure materials^5^ makes new opportunities for investigating novel thermoelectric devices with high efficiency^8^,^9^. Specially, spin caloritronics (spin Seebeck effect) was observed by Uchida et al^10^,^11^. They found that the spin-polarized currents (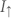 and 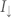) can be induced by a temperature gradient and flow in opposite directions. These wonderful discoveries strongly promote research on new energy of thermoelectricity^12^,^13^.

A single-molecule magnet (SMM) is a typical nanoscale material. In experiments, controlling the molecular spin^14^ and measuring thermopowers of molecule^15^,^16^,^17^,^18^,^19^,^20^ have been realised by directly using a scanning tunneling microscope. The spin-dependent transport properties, such as tunneling magnetoresistance(TMR) and spin Seebeck effect, were investigated in the sequential, cotunneling, and Kondo regimes using Wilson's numerical renormalization group and quantum master equation^21^,^22^,^23^,^24^,^25^. Many fantastic phenomena have been found in the experimental and theoretical studies, including negative differential conductance^26^,^27^, Berry phase blockade^28^, the magnetization of SMM controlled by spin-bias and thermal spin-transfer torque^29^. A SMM in a single spin state is necessary for generating the pure spin current^21^,^22^,^29^ without the magnetic field or magnetic electrodes. Meanwhile, it implies that the system temperature is limited by the blocking temperature of SMM (*T_B_*). When the symmetry of spin in the leads is broken, the angle (*θ*) between the easy axis of SMM and the spin orientation in the electrodes will influence the transport properties in the SMM devices. Specially, spin-bias^30^,^31^ and this angle (*θ*) are important and crucial on thermoelectric effect.

In this paper, we theoretically investigate the thermoelectric effects of a sandwich structure of NM/SMM/NM with spin-bias^29^,^32^,^33^ and angles (*θ*) between the easy axis of SMM and the spin orientation in the electrodes. We show that, in this system, pure spin currents are observed even though the system temperature is higher than the blocking temperature due to the spin symmetry broken by spin bias. In the linear regime, both thermopower and figure of merit are dependent on the angle and spin bias. It's worth noting that the angle plays a critical role on generating spin thermopowers. The figure of merit could tend to infinity by tuning the voltage gate at special angles, which implies that this system has an ultrahigh thermoelectric efficiency.

## Results

### Effective hamiltonian

The general Hamiltonian is expressed as^29^,^34^ *H* = *H_leads_* + *H_SMM_* + *H_t_*, in which
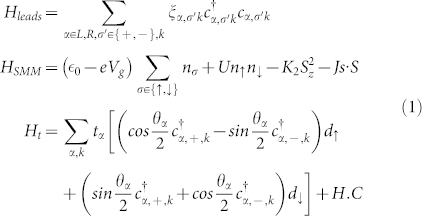
*H_leads_* describes the free electrons in two leads, with 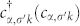 being the creation (annihilation) operator for a continuous state in the 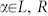 lead with the energy 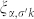 and spin index 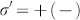, which denotes spin-majority (spin-minority) electrons. In this paper, wideband approximation is adopted and the density of states of the leads does not depend on the energy of the two leads. The chemical potential of *α* lead is defined as 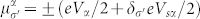 with 
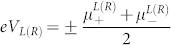
 and 
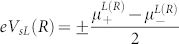
, and 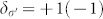 for 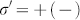. 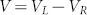 is the voltage and 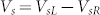 is the spin voltage. *P_α_* denotes the polarization of *α* lead and is defined as 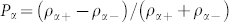. *H_SMM_* denotes the molecular degrees of freedom, in which 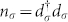 and 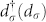 is the creation (annihilation) operators for the LUMO. 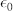 is the single-electron energy of the LUMO level, which is tuned by a gate voltage *V_g_*. U is the on-site Coulomb repulsion. *J* describes the Hund's rule coupling between the giant spin S of SMM and the electron spin in the LUMO, and parameter *K*_2_ is the easy-axis anisotropy of SMM. *H_t_* describes the tunneling between the LUMO of SMM and the electrodes, and *θ_α_* denotes the angle between the spin orientation of lead- *α* and the easy-axis of the SMM (as z-axis).

In the following, we turn to numerical calculations with parameters: *S* = 2, *J* = 0.1 *meV*, *K*_2_ = 0.04 *meV*, *U* = 1.0 *meV* and 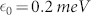. The tunneling parameters are set to 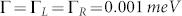. The properties of the leads are set to *P_R_* = *P_L_* = 0. Conventionally, *I_c_* and *I_s_* are defined as charge current and spin current respectively, and we set 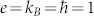 and the thermopower and current are scaled in the unit of 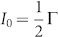. We can find all of the thermopowers are symmetric about *θ* = *π* because of the spatial symmetry of the sandwich structure.

### Transport properties

**Figure 1:**
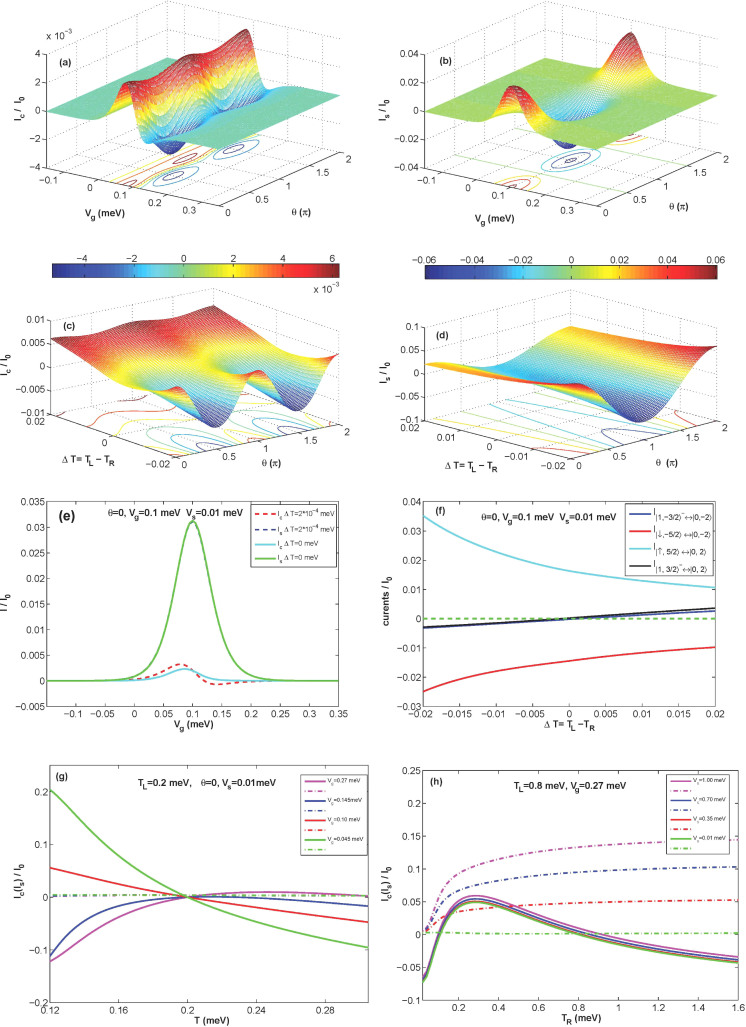
*I_c_* and *I_s_* as a function of *θ* for different *V_g_* and Δ*T* with parameters *S* = 2, 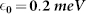, *J* = 0.1 *meV*, *K*_2_ = 0.04 *meV*, *U* = 1.0 *meV*, *k_B_* = 1, *P_L_* = *P_R_* = 0. In Fig (a) and (b), a tiny temperature difference is considered: 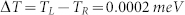 and *T* = 0.02 *meV*. In the (c) and (d), *V_g_* is set to 0.1 *meV* and the average temperature is fixed: *T* = 0.02 *meV*. (e) shows the temperature difference influences on the *I_c_* and *I_S_* with *θ* = 0. (f) displays the details of the constituents of the *I_c_* and *I_s_* with *θ* = 0. (g) displays *I_c_* and *I_s_* as a function of *T* for different *V_g_* with *T_L_* = 0.2 *meV* and *V_s_* = 0.01 *meV* at *θ* = 0. (h) shows *I_c_* and *I_S_* as a function of *T_R_* for different *V_s_* with *T_L_* = 0.8 *meV* and *V_g_* = 0.27 *meV* at *θ* = 0. Solid lines denote charge currents and dash dot lines mark spin currents in (g) and (h).

First, we consider that the left electrode is nonmagnetic with *V_s_* = 0.01 *meV* and *V* = 0 *meV*. The system temperature is lower than the anisotropy-induced energy barrier 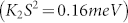. Figure 1a and Figure 1b show *I_c_* and *I_s_* as function of *θ* for different values of *V_g_* with Δ*T* = 0.0002 *meV*, respectively. In this case, we can find that *I_s_* is almost ten times of *I_c_*. The maximum or minimum value of *I_c_* and *I_s_* depends on the gate voltage *V_g_* and *θ*, but the positions of these extremums only depend on the *V_g_*. When 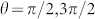, *I_s_* is exactly equal to zero due to the coefficient 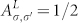, which leads 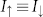. Moreover, we show *I_c_* and *I_s_* as a function of *V_g_* with two types of Δ*T* at *θ* = 0 in Figure 1e. One can find that *I_c_* is extremely sensitive to the temperature difference. However, *I_s_* only has a little change. Conventionally, the Fermi-Dirac distributions of spin-up and spin-down electron in the electrode are different due to finite *V_s_*. The higher the temperature is, the less these differences are generated.

In Figure1c and 1d, we show *I_c_* and *I_s_* as a function Δ*T* for different *θ* at *V_g_* = 0.1 *meV* and *T* = 0.02 *meV*, respectively. In this case, interesting phenomena can be observed. When *θ* = 0, *π*, 2*π*, *I_c_* first increases and then decreases with decreasing of Δ*T*, but it decreases monotonically when 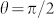. However, *I_s_* changes monotonically with decreasing Δ*T* and equals to zero at 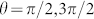.

### Thermoelectric coefficients

**Figure 2:**
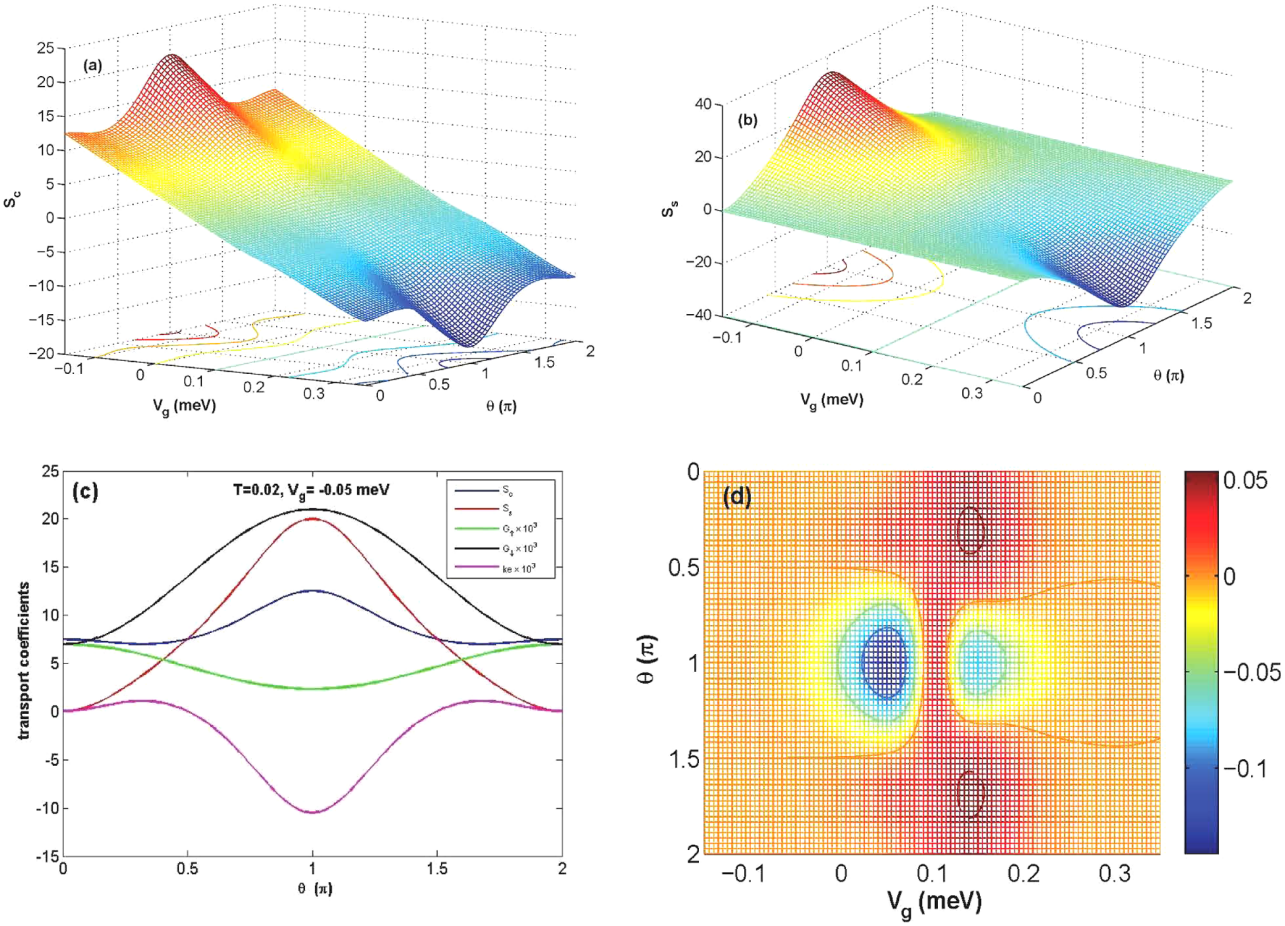
Here, we consider spin-bias only exists at the left lead. (a) and (b)show the *S_c_* and *S_s_* as a function of *θ* for different *V_g_* respectively. (c) displays the thermopowers as a function of *θ* with *V_g_* = −0.05 *meV* at *T* = 0.02 *meV*. (d) shows conventional thermal conductance (*k_e_*) as a function of *V_g_* and *θ* at *T* = 0.02 *meV*. The other parameters are chosen as same as that in Figure 1.

Next, we focus on the thermopower phenomena in the linear response regime and assume spin-bias only exists at the left electrode. Figure 2a and Figure 2b display charge-Seebeck and spin-Seebeck coefficient as a function of *θ* for different values of *V_g_* respectively. At the point *V_g_* = 0.1 *meV*, *S_c_* and *S_s_* are zero due to the same weight and the opposite transmission direction of the currents and energy carried by electrons and holes. At the special points *θ* = 0, 2*π*, the contributions of the spin-up and spin-down electrons are the same weight leading to 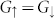 and *S_s_* = 0, no matter spin bias exists or not. *S_c_* and *S_s_* reach the maximum values for each *V_g_* when *θ* = *π*. In Figure 2c, we plot all transport coefficients as a function of *θ* with *V_g_* = −0.05 at *T* = 0.02. One can observe that electron thermal conductivity (*k_e_*) is zero at special *θ*, which may cause figure of merit *Z_c_T* or *Z_S_T* tend to be infinite. Furthermore, we plot the *k_e_* as function of *V_g_* and *θ* at *T* = 0.02 *meV* and find that zero *k_e_* exists only under special conditions of *V_g_* and *θ* in Figure 2d. It is interesting that one can have ultrahigh spin thermopower in a single molecular magnetism through manipulating the angle *θ* between the easy axis of SMM and the spin orientation of the electrodes, and also by tuning *V_g_*.

Thermoelectric coefficients have been investigated through solving the non-equilibrium Green's function (NEGF) in detail. The general formulas are derived to calculate the currents which depend on the angle *θ* between the easy axis of SMM and the spin orientation in the electrodes and spin bias. The spin bias destroys the SU(2) symmetry of electron-spin in nonmagnetic electrodes, which leads the fact that the angle *θ* influences the redistributions of different spin currents. It is amazing that pure spin currents can be obtained by tuning *V_g_* and Δ*T* with a finite spin bias and arbitrary *θ* except of 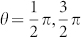. In the linear regime, infinite figure of merit can be generated by tuning *V_g_* at special angles *θ* with spin bias. Specially, when the angle *θ* is equal to zero or 2*π*, spin thermopowers vanish identically even though spin bias exists. These phenomenons may provide a new approach for the design of SMM devices based spin caloritronics.

## Discussion

The details of the constituents of the currents at *θ* = 0 are shown in Figure 1f. When *V_g_* is equal to 0.1 *meV*, the lowest-energy states of the isolated SMM are four-fold degenerate: 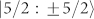 and 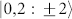, and the second-lowest-level state is a double degeneracy: 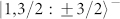. The energy level difference between the lowest and next-lowest levels is 0.0839 *meV*. The currents are mainly contributed by the transitions of 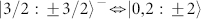 and 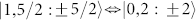. According to Eq. (9), we can approximate 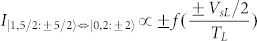 and 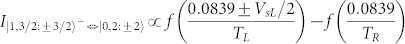. At the electron-hole symmetry point, 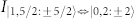 are only controlled by *V_sL_* and temperature *T_L_*.

However, 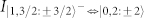 depend on the temperatures of the two leads, *V_sL_* and the energy-difference between the lowest and the second-lowest state. From Figure 1f, one can find that *I_s_* is mainly contributed by 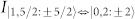, but *I_c_* is decided by all transitions. It is amazing that pure spin currents can be obtained at high temperatures. In Figure 1g, it is clear that *I_c_* first increases and then decreases with the increasing of the average temperature *T* at *V_g_* = 0.27 *meV*. But *I_S_* only has little change and is not equal to zero. Due to the spin splitting induced by spin bias, pure spin currents can be generated at arbitrary temperatures. In Figure 1h, the system temperature is five times as the anisotropy-induced energy barrier and pure spin currents can be obtained by increasing *V_sL_*.

Interestingly, the numerical results show *k_e_* can be equal to zero with changing *V_g_* and *θ* in Figure 2d. It means that *Z_s_T*(*Z_c_T*) may be infinite when *S_c_*(*S_s_*) and 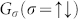 are finite. The exact choices of *V_g_* and *θ* are related to the details of the system's parameters. But it is necessary that *S_s_* must be larger than *S_c_*. It is well-known that electrons move from high temperature to low temperature, and it is not related to electronic spin. However, the spin-up and spin-down electrons under the spin bias can move in the opposite direction, and carry different energy according the Eq.10. Due to the competition between the temperature difference(Δ*T*) and spin bias (*V_sL_*), and the scattering between spin-up and spin-down electrons induced by the nonlinear spin exchange, it is possible that there are non-zero amount for thermopower and electrical conductance when thermal conductivity *k_e_* is zero.

## Methods

Non-equilibrium Hubbard Green function has been used to solve the thermoelectric transport in the sequential and linear response regime^35^. The system Hamilton can be rewritten by the transition operator^36^,^37^,^38^,^39^, i.e. 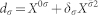 with 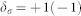 for 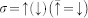, 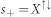, 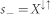, and 
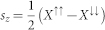
. For large spin, the operator can be expressed as^40^:
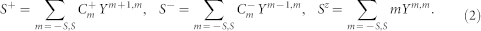
where *S* is the spin quantum number, 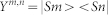 and 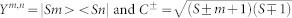. Finally, the retarded Green Function is written as 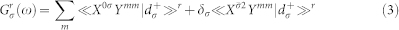
By using the Dyson equation and the Keldysh forum, the retarded(advanced) and the lesser(greater) Green's function can be compactly expressed as respectively 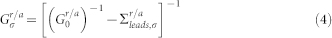

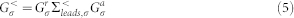


Here, 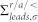
are the electron self-energy in the second-order approximation and the formulas for calculation are 
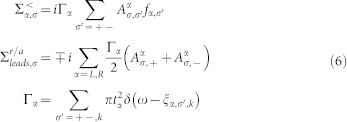
where
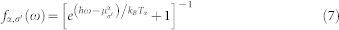

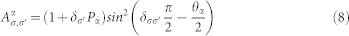


Here 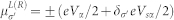 is a chemical potential with 
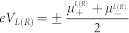
 and 
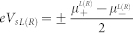
, and 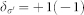
for 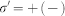. *P_α_* denotes the polarization of *α* lead and is defined as 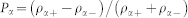. 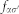 is the Fermi-Dirac distributions of *α* lead. 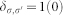 for 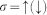 and 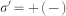

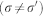
. The voltage is defined as 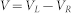
and the spin voltage is written as 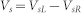.In the Hubbard operator representation, the eigenenergies of the unperturbed SMM can be obtained exactly, so the equation (5) is rigorous^41^. Following the Landauer-Buttikier formula, the expressions for the currents^42^ are
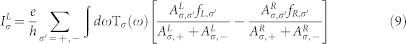

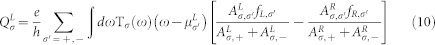
where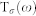 denotes the transmission coefficient of spin-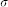 electrons
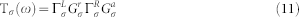
with
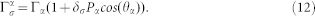
We can directly obtain the same formula for 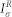 with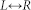, depicting electric current 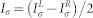 and heat current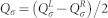. 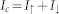 and 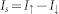 denote the charge current and spin current respectively. It is noticeable that our formulas (Eq. 9 and 10) are different from the conventional expressions^41^,^42^, in which *θ* and Fermi-Dirac function are coupled to each other.

In linear response regime, the thermoelectric coefficients are expressed as
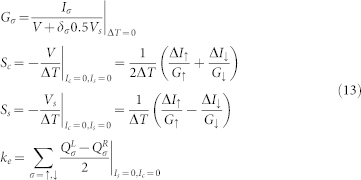
Here, *S_c_* and *S_s_* denote the charge Seebeck and spin Seebeck respectively. 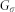 is 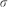
- electron conductance. *k_e_* is the conventional thermal conductance. 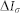
is the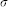-electric current induced by a temperature difference at zero voltage bias and zero spin bias. 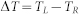 is defined as temperature difference and the average temperature is expressed as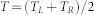. Finally, The spin figure of merit 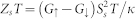 and charge figure of merit 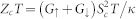 can be calculated. 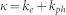
is the thermal conductance with contributions from both electrons
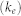
and phonons
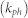
43,44,45. In our model, the phonon transport is not considered due to large mismatch of vibrational spectra between the SMM and leads.

## Author Contributions

K.Y. put forwards the idea and supervised the whole work. B.L. performed the numerical calculations and wrote the manuscript. B.L. and J.L. entered the discussions and analyzed the results. J.T.L. and J.H.G. made discussion on the referees' comments and revised the manuscript. All authors reviewed the manuscript.

## References

[b1] Dubi Y. & Di Ventra M. Colloquium: Heat flow and thermoelectricity in atomic and molecular junctions. Rev. Mod. Phys 83, 131–155 (2011).

[b2] Snyder G. J. & Toberer E. S. Complex thermoelectric materials. Nat Mater 7, 105–114, 1476–1122 (2008). 18219332 10.1038/nmat2090

[b3] Majumdar A. Thermoelectricity in semiconductor nanostructures. Science 303, 777–778, 0036–8075 (2004). 14764859 10.1126/science.1093164

[b4] Dubi Y. & Di Ventra M. Thermoelectric effects in nanoscale junctions. Nano Lett 9, 97–101 (2009). 19072125 10.1021/nl8025407

[b5] Dresselhaus M. S. *et al.* New Directions for Low-Dimensional Thermoelectric Materials. Adv Mater 19, 1043–1053, 1521–4095 (2007).

[b6] Garg A., Rasch D., Shimshoni E. & Rosch A. Large violation of the Wiedemann-Franz law in Luttinger liquids. Phys Rev Lett 103, 096402 (2009). 19792814 10.1103/PhysRevLett.103.096402

[b7] Kubala B., König J. & Pekola J. Violation of the Wiedemann-Franz law in a single-electron transistor. Phys Rev Lett 100, 066801 (2008). 18352503 10.1103/PhysRevLett.100.066801

[b8] Finch C., García-Suárez V. & Lambert C. Giant thermopower and figure of merit in single-molecule devices. Phys Rev B 79, 033405 (2009).

[b9] Rejec T., Žitko R., Mravlje J. & Ramšak A. Spin thermopower in interacting quantum dots. Phys Rev B 85, 085117 (2012).

[b10] Uchida K. *et al.* Observation of the spin Seebeck effect. Nature 455, 778–781, 0028-0836 (2008). 18843364 10.1038/nature07321

[b11] Uchida K. *et al.* Spin seebeck insulator. Nat Mater 9, 894–897 1476–1122 (2010). 20871606 10.1038/nmat2856

[b12] Ni Y. *et al.* The transport properties and new device design: the case of 6,6,12-graphyne nanoribbons. Nanoscale 5, 4468–4475 (2013). 23584607 10.1039/c3nr00731f

[b13] Ni Y. *et al.* Spin seebeck effect and thermal colossal magnetoresistance in graphene nanoribbon heterojunction. Sci Rep 3, 1380; 10.1038/srep01380 (2013). 23459307 10.1038/srep01380 PMC3587885

[b14] Parks J. J. *et al.* Mechanical control of spin states in spin-1 molecules and the underscreened Kondo effect. Science 328, 1370–1373, 1186874 (2010). 20538943 10.1126/science.1186874

[b15] Yee S. K., Malen J. A., Majumdar A. & Segalman R. A. Thermoelectricity in fullerene-metal heterojunctions. Nano Lett 11, 4089–4094 (2011). 21882860 10.1021/nl2014839

[b16] Evangeli C. *et al.* Engineering the thermopower of C60 molecular junctions. Nano Lett 13, 2141–2145 (2013). 23544957 10.1021/nl400579g

[b17] Guo S., Zhou G. & Tao N. Single molecule conductance, thermopower, and transition voltage. Nano Lett 13, 4326–4332 (2013). 23895464 10.1021/nl4021073

[b18] Liu J. *et al.* Ultralow thermal conductivity of atomic/molecular layer-deposited hybrid organic-inorganic zincone thin films. Nano Lett 13, 5594–5599 (2013). 24164650 10.1021/nl403244s

[b19] Reddy P., Jang S. Y., Segalman R. A. & Majumdar A. Thermoelectricity in molecular junctions. Science 315, 1568–1571 (2007). 17303718 10.1126/science.1137149

[b20] Aradhya S. V. V., Latha. Single-molecule junctions beyond electronic transport. Nat Nanotechnol 8, 399–410 (2013). 23736215 10.1038/nnano.2013.91

[b21] Wang R.-Q., Sheng L., Shen R., Wang B. & Xing D. Y. Thermoelectric Effect in Single-Molecule-Magnet Junctions. Phys Rev Lett 105, 057202 (2010). 20867951 10.1103/PhysRevLett.105.057202

[b22] Zhang Z., Jiang L., Wang R., Wang B. & Xing D. Y. Thermoelectric-induced spin currents in single-molecule magnet tunnel junctions. Appl Phys Lett 97, 242101(2010).

[b23] Cornaglia P. S., Usaj G. & Balseiro C. A. Tunable charge and spin Seebeck effects in magnetic molecular junctions. Phys Rev B 86, 041107 (2012).

[b24] Misiorny M., Weymann I. & Barnaś J. Interplay of the Kondo Effect and Spin-Polarized Transport in Magnetic Molecules, Adatoms, and Quantum Dots. Phys Rev Lett 106, 126602 (2011). 21517336 10.1103/PhysRevLett.106.126602

[b25] Misiorny M., Weymann I. & Barnaś J. Influence of magnetic anisotropy on the Kondo effect and spin-polarized transport through magnetic molecules, adatoms, and quantum dots. Phys Rev B 84, 035445 (2011). 10.1103/PhysRevLett.106.126602 21517336

[b26] Heersche H. *et al.* Electron Transport through Single Mn12 Molecular Magnets. Phys Rev Lett 96, 206801 (2006). 16803192 10.1103/PhysRevLett.96.206801

[b27] Misiorny M., Weymann I. & Barnaś J. Spin diode behavior in transport through single-molecule magnets. Europhys Lett 89, 18003 10295–15075 (2010).

[b28] González G. & Leuenberger M. N. Berry-phase blockade in single-molecule magnets. Phys Rev Lett 98, 256804 (2007). 17678045 10.1103/PhysRevLett.98.256804

[b29] Lu H.-Z., Zhou B. & Shen S.-Q. Spin-bias driven magnetization reversal and nondestructive detection in a single molecular magnet. Phys Rev B 79, 174419 (2009).

[b30] Lim J. S., López R., Limot L. & Simon P. Nonequilibrium spin-current detection with a single Kondo impurity. Phys Rev B 88, 165403 (2013).

[b31] Kovalev A. A. & Tserkovnyak Y. Thermomagnonic spin transfer and Peltier effects in insulating magnets. Europhys Lett 97, 67002 (2012).

[b32] Lu H.-Z. & Shen S.-Q. Using spin bias to manipulate and measure spin in quantum dots. Phys Rev B 77, 235309 (2008).

[b33] Xing Y., Sun Q.-f. & Wang J. Spin bias measurement based on a quantum point contact. Appl Phys Lett 93, 142107 (2008).

[b34] Elste F. & Timm C. Transport through anisotropic magnetic molecules with partially ferromagnetic leads: Spin-charge conversion and negative differential conductance. Phys Rev B 73, 235305 (2006).

[b35] Niu P.-B., Zhang Y.-Y., Wang Q. & Nie Y.-H. Quantum transport through anisotropic molecular magnets: Hubbard Green function approach. Phys Lett A 376, 1481–1488 (2012).

[b36] Fransson J. Non-equilibrium Nano-physics: A Many-body Approach. Vol. 809 (Springer, 2010).

[b37] Fransson J., Eriksson O. & Sandalov I. Many-Body Approach to Spin-Dependent Transport in Quantum Dot Systems. Phys Rev Lett 88, 226601 (2002). 12059440 10.1103/PhysRevLett.88.226601

[b38] Esposito M. & Galperin M. Transport in molecular states language: Generalized quantum master equation approach. Phys Rev B 79, 205303 (2009).

[b39] Kostyrko T. & Bułka B. Hubbard operators approach to the transport in molecular junctions. Phys Rev B 71, 235306 (2005).

[b40] Miyashita S. & Ogata M. Nagaoka ferromagnetism in large-spin fermionic and bosonic systems. Phys Rev B 80, 174422 (2009).

[b41] Ryndyk D. A., Gutiérrez R., Song B. & Cuniberti G. Green Function Techniques in the Treatment of Quantum Transport at the Molecular Scale. arXiv:0805.0628v2.

[b42] Haug H. & Jauho A. P. Quantum Kinetics in Transport and Optics of Semiconductors (ed. Klitzing, KV) (Springer Series in Solid-State Sciences, Springer, Berlin, 1996).

[b43] Mingo N. Anharmonic phonon flow through molecular-sized junctions. Phys Rev B 74, 125402 (2006).

[b44] Wang J.-S., Wang J. & Zeng N. Nonequilibrium Green's function approach to mesoscopic thermal transport. Phys Rev B 74, 033408 (2006).

[b45] Yamamoto T. & Watanabe K. Nonequilibrium Green's Function Approach to Phonon Transport in Defective Carbon Nanotubes. Phys Rev Lett 96, 255503 (2006). 16907319 10.1103/PhysRevLett.96.255503

